# Does Melanoma Begin in a Melanocyte Stem Cell?

**DOI:** 10.1155/2012/571087

**Published:** 2012-12-18

**Authors:** James D. Hoerter, Patrick Bradley, Alexandria Casillas, Danielle Chambers, Brandon Weiswasser, Lauren Clements, Sarah Gilbert, Albert Jiao

**Affiliations:** Department of Biological Sciences, Ferris State University, Big Rapids, MI 49307, USA

## Abstract

What is the cellular origin of melanoma? What role do melanocyte stem cells (MSC) and other melanocyte precursors play in the development of melanoma? Are MSCs and other latent melanocyte precursors more susceptible to solar radiation? These and many other questions can be very effectively addressed using the zebrafish model. Zebrafish have a robust regenerative capability, permitting the study of how MSCs are regulated and recruited at specific times and places to generate the pigment pattern following fin amputation or melanocyte ablation. They can be used to determine the effects of environmental radiation on the proliferation, survival, repair, and differentiation of MSCs. Our lab is using zebrafish to investigate how UVA- (320–400 nm) and UVB- (290–320 nm) induced damage to MSCs may contribute to the development of melanoma. A review is given of MSCs in zebrafish as well as experimental techniques and drugs for manipulating MSC populations. These techniques can be used to design experiments to help answer many questions regarding the role of MSCs or melanocyte precursors in the formation of melanoma stem cells and tumors following exposure to UVA/UVB radiation.

## 1. Introduction

*Danio rerio*, a small tropical fish known commonly as zebrafish, is becoming a widely used model system for studying many human diseases [1]. Zebrafish has a wealth of practical benefits as a laboratory model system. The cost of husbandry is low and large numbers of fish are easy to maintain for experimental observation [2–4]. Their high degree of genetic similarity to humans assures that experimental findings are highly relevant to human disease and cancer pathways.

### 1.1. UV Radiation and Melanoma

Zebrafish has great promise as a model for investigating the earliest origins of melanoma [5]. Although it is well established that solar ultraviolet radiation is involved in the etiology of melanoma, the relative role of solar ultraviolet-B (UVB; 290–320 nm) and ultraviolet-A (UVA; 320–400 nm) radiation in its initiation, promotion and progression are still not clear [6]. Decades of epidemiological studies have linked solar UV radiation to human skin cancers [7]. The specific contribution of UVB and UVA radiation exposure in the risk of melanoma is controversial. There is adequate evidence to suggest that both UVA and UVB radiation act together to promote the development and progression of malignant melanoma, but how each wavelength contributes to this process is still controversial. Because the UVB wavelengths are within a major portion of the DNA absorption spectrum, UVB can directly cause DNA damage. Longer-term effects such as persistent genomic instability and bystander effects have also been observed following UVB treatment [8–10]. On the other hand, UVA wavelengths penetrate the human skin more effectively than UVB wavelengths. More than 20% of UVA radiation reaches the basal layers of the skin compared to less than 10% of UVB radiation [11]. In contrast to UVB radiation which acts directly on DNA, UVA radiation acts indirectly through the production of reactive oxygen species that can damage major biomolecules including DNA, membrane lipids, and proteins. Damage to these molecules leads to significant biological effects including cytotoxicity, mutations, and alterations in cell signaling pathways [12].

### 1.2. The Melanocyte Stem Cell (MSC) and Melanoma

**Figure 1:**
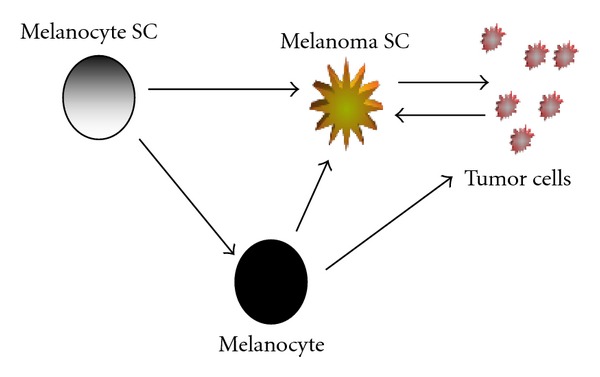
Possible transformation pathways in the development of melanoma. Schematic showing normal development and regeneration of a melanocyte proceeding from a melanocyte stem cell (SC) in the dermis of the skin. Mature melanocytes can be transformed to either a melanoma SC and/or directly to a tumor cell. Melanoma SCs may also form from a small subpopulation of cells comprising the tumor.

Traditionally, it has been assumed that melanoma has its earliest beginnings in a mature melanocyte, but due to the accumulating evidence that early-stage precursors to melanocytes exist in the dermis [13–17], equal attention is now being given to the hypothesis that cutaneous melanoma may also have its earliest origins in an extrafollicular MSC [18–20]. It is very probable that melanoma stem cells originate either from a transformed melanocyte, directly from a transformed MSC, or from a combination of both sources (Figure 1). Understanding the pathways controlling self-renewal, expansion, and differentiation of MSCs and how UVA and UVB radiation alters and disrupts these pathways will bring greater insight into origins of this deadly disease.

### 1.3. DNA Damage and Repair

UV light causes damage when DNA and proteins directly absorb the light energy or when it produces free radicals, including reactive oxygen species, which indirectly cause damage to these molecules. UV light induces cyclobutane pyrimidine dimers (CPDs) and pyrimidine (6-4) pyrimidone photoproducts (PDs). Generation of these photoproducts is wavelength dependent with UVB radiation being 1000-fold more efficient than UVA in their production. These two photoproducts can block DNA replication, transcription and affect protein-DNA interactions. If the photoproducts are improperly repaired or escape repair processes they cause mutations contributing to the development of melanoma [21–23]. In humans, CPDs and (6-4) PDs are removed from DNA through nucleotide excision repair (NER). In addition to NER, zebrafish also have photoenzymatic repair (PER) which utilizes photolyase to repair cyclobutane pyrimidine dimers formed between adjacent pyrimidines in UV-irradiated DNA. This light-driven enzymatic catalysis of DNA repair, known as photoreactivation, uses the energy in the visible light spectrum [24, 25]. This is a very fast and efficient process as illustrated in the *Xiphophorus* hybrid model of melanoma that removes 50–85% of the CPDs within 15 min and (6-4) PDs within 60 min [26, 27]. This difference in repair capability is an important distinction between humans and zebrafish, and precautions must be taken to reduce PER by keeping fish in the dark and minimizing exposure to light when making observations. Our lab is now exploiting the technique of *in vivo* electroporation to introduce a fluorescein-tagged antisense morpholino to block expression of photolyase and prevent PER in the regenerating tail fin [28]. Thus, the response to DNA damage is then more closely aligned to that which is found in human cells.

The significance of NER in preventing melanoma is illustrated in the inherited human condition xeroderma pigmentosum (XP). People homozygous for this recessive mutation have defective NER. Unable to repair DNA damage effectively, they develop skin tumors with high frequency in sun-exposed areas of the skin [29, 30]. Age-related loss of DNA damage-repair pathways due to UV-induced mutations may pose a significant threat to stem cell survival and function. Normal stem cells have strict control of gene expression and DNA replication whereas MSCs with compromised DNA repair pathways may have altered patterns of proliferation, quiescence, and differentiation, and may be more susceptible to malignant transformation [31]. In zebrafish, *p53* mutants are deficient in NER and are more sensitive than wild-type fish to UVB radiation [32]. Many studies show that *p53* gene expression is developmentally regulated and its expression depends upon the stage of development [32–36]. Thus, *p53* may play an important role in DNA damage repair in MSCs similar to what has been shown in other cells [37]. 

## 2. The Zebrafish Model

The zebrafish model lends itself well to testing the hypothesis that UV-induced damage to MSCs rather than to mature melanocytes may be how melanoma begins. UVA penetrates deeper in the dermal layer of the skin where MSC niches may reside [11]. UVA fingerprint mutations are most abundant in the basal germinative layer, suggesting that UVA- rather than UVB-induced DNA damage is an important carcinogen in the stem cell compartment of the skin [38]. MSCs residing in the dermis will accumulate DNA damage over the lifetime of an individual when protective and repair mechanisms are impaired due to the cumulative effects of UVA exposure from the sun or from indoor tanning beds that have much higher spectral irradiances than the sun [39]. How long mutated MSCs remain dormant will depend on the need of the skin to replace damaged cutaneous melanocytes. When an MSC is activated to replace a damaged melanocyte, it may take several months for a precursor to progress through all the stages leading to a fully differentiated melanocyte on the basal layer of the epidermis [40]. As a melanocyte precursor moves through the various stages of differentiation it may become more vulnerable to the damaging effects of UV radiation.

The zebrafish model offers a variety of strategies to determine if UVA and UVB radiation impairs MSC function simply by activating melanocyte regeneration by either fin amputation or chemical ablation of the adult melanocyte population [41–45]. Any mutations affecting signaling pathways controlling migration, proliferation, or differentiation of the melanocyte from these precursors will be reflected in the regenerated pigment pattern. In our lab, we are investigating how long-term exposure to UVA/UVB radiation impairs pathways regulating MSC function, contributing to increased proliferation and abnormal migration of melanocytes. Our ultimate goal is to identify a specific exposure regimen of UVA/UVB radiation that will significantly increase the incidence of melanoma in zebrafish. This will then permit us to design experiments to determine how irradiance, dose, and mode of exposure affect the incidence and frequency of tumors. It will also help to identify the origins of melanoma stem cells, and find markers for early detection and therapeutic intervention to eradicate the disease and prevent its recurrence [46]. 

Zebrafish MSCs exhibit almost unlimited self-renewal capacity and share many regulatory signals and pathways with melanoma and cancer stem cells in humans [47–49]. Genes controlling melanocyte development and tissue regeneration are known to play important roles in the development of melanoma. In summary, the zebrafish model has great potential for understanding how MSCs are affected by solar radiation, and what role they may play in the formation of melanoma stem cells and melanocyte tumors.

## 3. Melanocytes

There are two distinct populations of melanocytes in zebrafish—larval melanocytes and adult melanocytes. During embryonic development neural crest cells differentiate directly into early larval melanocytes. At 3 days after fertilization (dpf) there are approximately 460 larval melanocytes [50]. A “pigment pattern metamorphosis” occurs when the larval pigment pattern is replaced with the adult pigment pattern, culminating at 4-5 weeks after fertilization [51]. The adult melanocytes migrate and congregate to form the characteristic stripe patterns on the lateral surface of the body and fins. Over 250 mutations have been isolated affecting pathways controlling the development of pigment patterns. These mutations provide an excellent resource of genetic tools to dissect the cellular and molecular pathways regulating the development of larval and adult melanocytes [52]. The current model is that larval and adult melanocytes have different genetic requirements; larval melanocytes develop directly from the neural crest, whereas adult melanocytes develop from latent stem cells of presumptive neural crest origin [51, 53].

## 4. Melanocyte Precursors and Stem Cells

Cells of the melanocyte lineage that have not yet differentiated into a melanin-producing melanocyte are called melanoblasts, melanocyte progenitors, or latent melanocyte precursors. They all represent cells in a pre-melanocyte stage, somewhere along the developmental pathway that ends with differentiation into a mature, melanin-producing melanocyte. In contrast, a MSC has both the capacity for producing differentiated melanocyte progeny as well as the ability to self-renew. The existence of self-renewing MSCs in zebrafish is supported by studies showing continued regeneration of the melanocyte pigment pattern in fish following multiple rounds of adult melanocyte ablation or fin amputation [42, 43, 53, 54].

The cloning and expression of an early zebrafish melanoblast marker, dopachrome tautomerase (*dct*), permits the detection of melanoblasts very early in development. Analysis of *dct *expression suggests that melanoblast specification may be established prior to migration at about 18-19 hpf [55]. Melanoblasts migrate out along the axis of the embryo and differentiate into melanocytes at approximately 24 hours hpf. Niches for these precursors are associated with peripheral nerves and ganglia, including dorsal root ganglia, ventral motor root fibers, lateral line nerves, and nerve fibers coursing through the myotomes. This is consistent with studies in mice where Schwann cell precursors are thought to be the cellular origin of melanocytes [56, 57], demonstrating the conservation of developmental processes across large evolutionary distances.

Pools of quiescent or dormant precursors persist in the adult, and different pools have different capacities for regenerating melanocytes; precursor pools for late metamorphic, hypodermal melanocytes persist in the adult yet have a finite regenerative potential, whereas precursor pools for adult scale melanocytes are more highly regulated and have a greater regenerative potential. This suggests that spatially and temporally distinct pools of melanocyte precursors have different morphogenetic and differentiative potentials [51]. 

## 5. Melanocyte Regeneration

The zebrafish model can be used to investigate how adult stem cells may be more susceptible to environmental radiation at different stages of development. In general, when tissues are regenerated, adult stem cells switch from a quiescent stage and proceed through all the intermediate stages leading up to fully differentiated cell types. A major challenge of using stem cells *in vitro* for testing the effects of environmental radiation is that they do not take into consideration the key roles that intercellular communication and homeostasis play in how a cell responds and adapts to the radiation. The MSC has a tractable lineage; it is easy to document changes in tissue organization (pigment pattern) and proliferation (tumors and nevi) over the lifetime of the fish. Exposing the MSCs at specific stages of development for extended periods of time can be more precisely achieved by adding WNT pathway inhibitors to temporarily suspend regeneration while irradiating [58]. 

The amputation of the zebrafish caudal fin is a very facile method to initiate and synchronize the regeneration of melanocytes from the resident MSC population. Amputation of the caudal fin does not affect fish viability. Fin regeneration is completed within two weeks. The caudal fin has an almost unlimited capacity to regenerate; repeated amputations up to 29 times over a period of 11 months do not alter regenerative capacity [54, 59]. During regeneration of the caudal fin, melanocyte pigment pattern is restored through the activation of resident MSCs and not from the migration of previously differentiated melanocytes [53]. Thus, any abnormalities in melanocyte proliferation or pigment pattern in regenerated fins following exposure to UVA/UVB radiation suggest DNA lesions in the MSCs and/or alterations in epigenetic pathways. Leading-edge MSCs in the regenerating fin divide asymmetrically to generate adult melanocytes and symmetrically to expand the MSC pool. This ensures that each fin ray has at least one MSC to regenerate new melanocytes as the need arises [60]. This feature provides a powerful method to study how UVA/UVB radiation alters the switches that control both symmetric and asymmetric division of stem cells. This can be accomplished by selectively exposing only the leading edge of the regenerating fin and observing for abnormalities in melanocyte proliferation and pigmentation in the fin.

Although the fin amputation offers many advantages to study the mechanisms regulating MSC proliferation and their distribution in regenerating tissue, this method does not offer the opportunity to study the effects of UVA/UVB radiation on MSCs in isolation from other biological mechanisms operating during epimorphic regeneration. This limitation can be overcome by using the conditional drug, neocuproine (NCP), to ablate the entire population of adult melanocytes [42] or by using a laser to destroy a single adult melanocyte [61]. Thus, the regeneration of melanocyte populations from resident adult MSCs can be studied in isolation of other biological mechanisms operating during epimorphic regeneration of the fin. 

## 6. Genes and Signaling Pathways

Zebrafish mutants help to delineate the mechanisms, underlying the establishment, maintenance and recruitment of MSCs or melanocyte precursors during development and regeneration [62]. The* KIT*, *RAS*, *MITF*, and *ERBB3 *genes and the *Wnt*/*β*-catenin signaling pathway are known to play important roles in the development and regulation of MSCs or melanocyte precursors. 

### 6.1. *KIT* (Tyrosine-Protein Kinase)

Tyrosine-protein kinase is encoded by the *KIT* gene [63]. When this receptor binds to stem cell factor (SCF) it forms a dimer that activates its intrinsic tyrosine kinase activity, that in turn phosphorylates and activates signal transduction molecules that propagate the signal in the cell. *KIT* signaling influences the establishment, recruitment, regulation, and maintenance of both the pre-MSC and the MSC population itself [62, 64, 65]. Melanoblasts express the *KIT *receptor, and it is believed that SCF guides these cells to their terminal locations. SCF also regulates survival and proliferation of fully differentiated melanocytes in adults [66]. Mutants in the KIT receptor fail in the production of late-developing melanocytes of the lateral stripe. The increased expression of *KIT* in transgenic larvae leads to recruitment of MSCs, bringing about an increased proliferation of melanocytes. This suggests that *KIT* signaling is involved in MSC recruitment during regeneration [65].

### 6.2. RAS

*RAS* proteins function as binary molecular switches that control intracellular signaling networks. *RAS*-regulated signaling pathways control such processes as actin cytoskeletal integrity, proliferation, differentiation, cell adhesion, apoptosis, and cell migration. *RAS* proteins are often deregulated in cancers, leading to increased invasion and metastasis and decreased apoptosis. *RAS* determines the supply of melanocyte precursors, facilitating stripe pattern recovery during zebrafish fin regeneration. During regeneration following amputation of the caudal fin, *RAS *activity levels control the repopulation and expansion of the MSC or melanocyte precursor cell population that exists prior to amputation, rather than amplifying existing differentiated melanocytes. The level of active *RAS* in regenerating zebrafish fins controls the density of melanocyte precursors and differentiated progeny within newly forming fin structures. Transgenic increases in active *RAS* markedly amplify the number of melanocytes present during fin regeneration, hyperpigmenting new fin structures and disrupting the stripe pattern. The amplification of melanocyte precursors occurs through a mechanism that does not depend on intact *KIT* signaling [41]. Expression of oncogenic *HRAS* under the *KIT* promoter induces melanoma development with high efficiency without the need for additional mutations in tumor suppressor genes [46].

### 6.3. *MIT* (Microphthalmia-Associated Transcription Factor)

The *MIT* gene encodes the microphthalmia-associated transcription factor, a protein of the basic helix-loop-helix leucine zipper family. It is the master regulator of vertebrate melanocyte development [67–69]. It is required for the development and survival of direct-developing embryonic (ontogenetic) progenitors and adult melanocytes but does not appear to be required for establishment or survival of the MSC population in the adult [64, 70, 71]. *MITF* promotes melanoblast survival immediately after migration from the dorsal neural tube and may also directly or indirectly affect the rate at which melanoblast number increases during dorsolateral pathway migration [72]. Activation of *MITF *expression is thought to be a key event in triggering MSCs to enter the cell cycle [73]. 

### 6.4. *ERBB3* (Receptor Tyrosine-Protein Kinase)

The *ERBB3* gene encodes a member of the epidermal growth factor receptor (EGFR) family of receptor tyrosine kinases. This membrane-bound protein has a neuregulin binding domain but not an active kinase domain. Therefore, it can bind this ligand but not convey the signal into the cell through protein phosphorylation. However, it does form heterodimers with other EGF receptor family members which do have kinase activity. This leads to the activation of cell proliferation or differentiation pathways [74, 75]. *ERBB3* is required during embryonic development for establishing the pool of melanocyte precursors responsible for developing adult melanocytes during the transition from the larval to adult stage. This is in contrast to embryonic melanocytes that develop directly, without the need for *ERBB3* signaling [64, 65, 76]. Nerve-associated melanocyte precursors are missing or reduced in the *ERBB3-*deficient fish [51]. 

### 6.5. *Wnt/*β**-Catenin Signaling Pathway

The *Wnt/*β**-catenin signaling pathway plays a variety of important roles in embryonic development, cell differentiation, and regulation of stem cells [77, 78]. It is a key pathway involved in controlling stem cell activation during regeneration [79, 80]. Pathological states that may arise from altered stem cell function, such as degenerative diseases and cancer, are frequently associated with changes in the *Wnt/*β**-catenin pathway [58]. *Wnt/*β**-catenin signaling is activated during regeneration of the zebrafish caudal fin. It is required for the formation and subsequent proliferation of the progenitor cells in the blastema of the regenerating fin [81].

## 7. Drugs Regulating MSCs

Several chemicals offer pharmacological methods to prevent development or activate MSC pathways. AG1478 prevents progression of a pre-MSC [76], ICI-118,551 prevents progression of a post-MSC [65], and IWR-1 temporarily halts progression of a MSC into a differentiated melanocyte by suspending regeneration [58]. NCP, a copper chelator, ablates the entire adult melanocyte population and upon washout, initiates melanocyte regeneration from resident MSCs [42]. MoTP specifically ablates larval melanocytes or melanoblasts before 6-7 dpf, thereby activating regeneration of larval melanocytes from the resident MSC population. Artificially regulating and manipulating the development of specific stages of melanocyte development *in vivo* create “experimental windows” to assess effects of radiation and chemicals on specific melanocyte precursor populations. Alternations are manifested by abnormal pigment patterns or melanocyte proliferation (nevi and tumors).

### 7.1. AG1478

AG 178 is an epidermal growth factor receptor tyrosine kinase inhibitor of the *ERBB* family of receptors [82]. When this drug is applied during early embryonic development (9–48 hpf), no differences are observed in the number of melanocytes that develop in either the trunk or the tail. However, larvae lack the ability to regenerate melanocytes after ablation. The most dramatic effect is seen after metamorphosis, when the AG1478-treated fish fail to develop adult melanocytes. Several lines of evidence suggest that AG1478 exerts its effect by preventing a pre-MSC from progressing to a MSC. This results in a MSC that is not responsive to regulative signals required for the development of adult melanocytes during metamorphosis [65]. 

### 7.2. ICI-118,551

The regeneration specific drug, ICI-118,551, is a small molecule that blocks melanocyte regeneration in larvae by preventing the developmental progression of a MSC to the melanoblast stage. The melanoblasts express dopachrome tautomerase (*dct* ^+^), an important regulatory enzyme that plays a pivotal role in the biosynthesis of melanin, but never develop into fully differentiated melanocytes that express melanin [65].

### 7.3. Neocuproine (NCP)

Neocuproine (NCP), when added to fish water, triggers apoptosis of the adult melanocyte population in zebrafish. It has no effect on either larval melanocytes or the resident MSC population. After drug washout, the pigment pattern is regenerated from resident MSCs within 4 weeks [42]. Thus, the regeneration of a single cell type from a resident adult stem cell population, in this case the MSCs, can be studied in isolation of other biological mechanisms operating during epimorphic regeneration of a fin. This drug provides a powerful tool for ablating adult melanocytes and exploring the role of MSCs in the maintenance and regeneration of melanocytes. It also offers the opportunity to selectively expose MSCs but not adult melanocytes to various dosages and irradiances of UVA/UVB radiation.

### 7.4. MoTP

This small pro-drug molecule, (2-morpholinobutyl)-4-thiophenol (MoTP), specifically ablates larval melanocytes or melanoblasts when administered before 6-7 dpf or at 15 dpf [83]. Its cytoxicity to melanocytes is created when tyrosinase converts the drug to a cytotoxic quinone species. Upon melanocyte ablation by MoTP treatment, MSCs are released from their quiescent state, divide, and amplify via melanoblasts to regenerate new melanocytes and reestablish the pigment pattern. Thus, MSCs can be induced to reenter developmental pathways and reconstitute the larval melanocyte population following melanocyte ablation by simply treating with MoTP. This is achieved by incubating zebrafish embryos in MoTP up to 6-7 dpf to ablate melanoblasts or melanocytes. By 72 hpf, when untreated larvae have established their melanocyte pattern, MoTP-treated larvae are devoid of all melanocytes. When these larvae are transferred to MoTP-free water at 72 hpf, MSCs gradually regenerate melanocytes over the next 4-5 days. This is a very effective tool to initiate melanocyte regeneration from MSCs. It is estimated that as few as 15, or as many as 145, quiescent MSCs in the zebrafish larvae give rise to the approximately 350–400 melanocytes at the completion of regeneration [83]. MoTP is not effective in adult fish except at the onset of metamorphosis. Thus, MoTP has limitations for activating MSC populations because it can only be used during a very limited time period (before 6-7 dpf or at 15 dpf) and can only be done once during the lifetime of the fish [42]. Despite these limitations, environmental exposure to radiation can be evaluated for their effects on MSC populations at these times by treating with MoTP and then observing for abnormalities in pigment pattern. 

### 7.5. IWR-1

This small-molecule inhibitor acts as a negative regulator of canonical *Wnt* signaling [58]. *Wnt/*β**-catenin signaling is important in the activation of stem cells in heart, muscle, bone and liver [79, 80, 84, 85]. *Wnt/*β**-catenin signaling regulates blastema formation and subsequent proliferation during regeneration of the caudal fin following amputation. IWR will temporarily stop regeneration of the caudal fin at any stage. It can be used to suspend the progression of MSC into mature melanocytes at any stage just by varying the time at which the chemical is added to the water. Fish maintained in water containing IWR-1 for 7 days are able to resume tissue regeneration to normal levels after IWR-1 washout, which suggests that transient inhibition of *Wnt/B*-catenin response does not permanently alter the ability of MSCs to self-renew and participate in the regeneration of melanocytes [58, 86]. Inhibiting *Wnt* signaling with IWR provides a powerful method to temporarily suspend developmental progression of a MSC at any period of time or stage, creating “experimental windows” to subject MSCs and melanocyte precursors to radiation. 

## 8. Applications

The zebrafish model is becoming more widely used for melanoma research [87]. Several studies have provided the groundwork for exploring the role of UV light in the development of melanoma in zebrafish. For example, it has been found that expression of the BRAF^V600E^ mutation, the most common mutation in human nevi and melanoma, is sufficient to induce ectopic nevi in zebrafish and can collaborate with *p53* mutations to promote melanoma [88, 89]. It has been discovered that zebrafish have a competent *p53*-dependent NER pathway to protect cells from UVB-induced DNA damage in the skin [32]. We are using the zebrafish model in our lab to determine the effects of UVA/UVB radiation on the MSC population. These studies are providing a better understanding of the early events contributing to the development of melanoma. By using multiple rounds of melanocyte ablation or fin amputation to induce melanocyte regeneration from MSCs, we are assessing whether the UVA/UVB wavelengths in solar radiation can alter fundamental activities operating in stem cells such as DNA repair capacity, fate determination, and function. 

**Figure 2:**
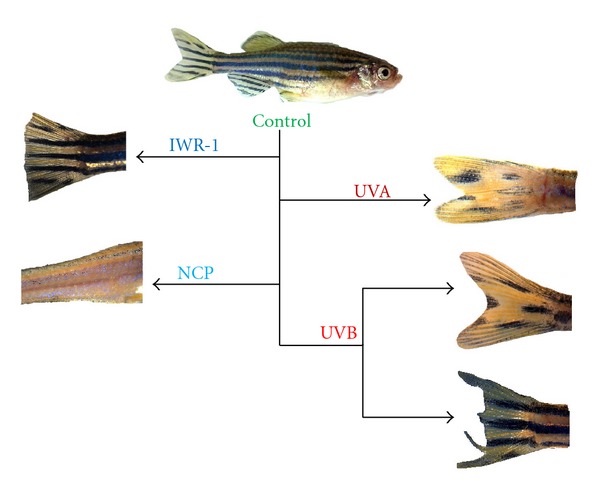
Methods for initiating, suspending, or altering melanocyte regeneration from MSCs. Top: representative wild-type zebrafish pigment pattern. Left: representative examples of fish treated with IWR-1 and NCP; caudal fin regeneration is delayed with IWR-1, melanocytes ablated with NCP (lateral stripe is shown after treatment). Right: representative examples of fish exposed to UVA or UVB radiation. UVA irradiation following NCP treatment impairs MSCs function, altering pigment pattern; UVB irradiation before fin amputation impairs MSCs function, altering pigment pattern or disrupts epimorphic regeneration of caudal fin.

We use IWR-1 to “freeze” melanocyte precursors at specific times of development to discover stages in the MSC developmental pathway where a melanocyte precursor may be more vulnerable to the damaging rays of UVA or UVB radiation (Figure 2). Our preliminary findings suggest that this radiation is indeed capable of impairing MSC function as reflected in abnormal regeneration of pigment pattern. UVB radiation is also capable of impairing stem cell function responsible for epimorphic regeneration as reflected in abnormal fin morphology or termination of fin outgrowth.

## 9. Conclusion

The zebrafish model has significant potential for discovering how UV-induced damage to MSCs may play a role in the development of melanoma. It offers many experimental approaches for identifying the many important variables in the initiation, development, and progression of melanoma. Variables include fluence, UV spectra, and dosage. In a general way, the zebrafish model has great potential for assessing the effects of any chemical on the function, proliferation, and differentiation of adult stem cells. This may have major implications for understanding the overall role of stem cells in the genesis of tumors or as precursors of many degenerative diseases in humans.
